# Effect of daphnetin combined with tobramycin on *Pseudomonas aeruginosa* biofilm infection *in vitro* and *in vivo*

**DOI:** 10.3389/fimmu.2025.1648096

**Published:** 2025-08-08

**Authors:** Dingbin Li, Chao Mao, Siyuan Chen, Zhongwei Wang, Weilin Zhang, Zhencong Li, Liangsheng Li, Chaoqin He, Weixiong Guo, Jinsong Wei, Qingjun Wei

**Affiliations:** ^1^ Department of Spinal Degeneration and Deformity Surgery, Affiliated Hospital of Guangdong Medical University, Zhanjiang, Guangdong, China; ^2^ Department of Orthopedics, The Second Affiliated Hospital of Guangxi Medical University, Nanning, Guangxi, China

**Keywords:** daphnetin, biofilm, tobramycin, *Pseudomonas aeruginosa*, combination

## Abstract

**Objective:**

The aim of this study was to investigate the effectiveness of daphnetin in combination with tobramycin on *Pseudomonas aeruginosa* biofilm infection *in vitro* and *in vivo*.

**Method:**

The study was divided into four groups: control, tobramycin, daphnetin, and tobramycin combined with daphnetin groups. First, a 72-h *Pseudomonas aeruginosa* biofilm model was established *in vitro*. The antibacterial effects of daphnetin and tobramycin alone and in combination were evaluated using various methods, including microdilution, crystal violet staining, colony counting, and electron microscopy. Then, a model of *Pseudomonas aeruginosa* biofilm infection in rabbit joints was established *in vivo*. After 7 days of continuous treatment, the rabbits were sacrificed on day 14 post infection. The therapeutic effect of daphnetin and/or tobramycin was further evaluated by observing the gross anatomy of the knee joint, biofilm PNA-FISH, synovial bacterial load, and pathology.

**Results:**

The results showed that daphnetin had a minimum inhibition concentration (MIC) of 890 µg/mL against the PAO1 strain, while tobramycin had an MIC of 2.75 µg/mL against the same strain. Crystal violet staining and colony counting showed that the biofilm in the group treated with both daphnetin and tobramycin was significantly less than that in the control group (P < 0.05). Scanning electron microscopy further confirmed that the combination of daphnetin and tobramycin had the strongest bactericidal effect. *In vivo*, the knee joint of rabbits in the daphnetin combined with tobramycin group had the least gross anatomical inflammatory response, amount of PNA-FISH biofilm, synovial colony count, synovial pathological examination of inflammatory cell infiltration, and synovial thickening.

**Conclusion:**

The study indicated that daphnetin may be a promising synergist that enhances the activity of tobramycin against *Pseudomonas aeruginosa* biofilm infection *in vitro* and *in vivo*.

## 1 Introduction

Suppurative arthritis (SA) is a condition in which an infection in the joint space destroys the articular cartilage, causes synovitis, and impairs joint function (1, 2). Current treatments for SA include antibiotic therapy and surgery (3). However, bacterial resistance poses a challenge to the treatment of SA, and antibiotic tolerance is one of the reasons for drug resistance. Pathogenic bacteria can develop antibiotic tolerance through mechanisms such as biofilm (BF) formation (4, 5). *Pseudomonas aeruginosa* (PA) is one of the major bacteria responsible for nosocomial infections in SA (6).

PA is an opportunistic gram-negative pathogen that can thrive in different ecological environments and hospital conditions because of its extraordinary metabolic diversity, genomic plasticity, resistance to environmental stress, natural resistance to antibiotics, strong ability to form BF, and quorum sensing (QS) systems that regulate the expression of virulence factors (7, 8). The World Health Organization has classified PA as a “key priority pathogen” because of its resistance to broad-spectrum antibiotics, including third-generation cephalosporins and carbapenems (9, 10). PA has two survival modes: planktonic bacteria and BF. PA-BF is a microbial aggregate formed by PA adhering to the surface of the medium through its secreted extracellular polymeric matrix and enveloping itself (11). It is known that more than 80% of PA infections are associated with PA-BF (12). Once formed, PA-BF reduces immune cell and antibiotic damage to bacteria, leading to clinically refractory infections (13). One literature report indicated that the multidrug resistance rate of PA was 88.3%, among which 30.1% were extensively drug-resistant. The resistance rates to ceftazidime and ciprofloxacin were 75.0% and 46.6%, respectively (14).

BF formation has been reported in keratitis (15), empyema (16), tonsillitis (17), and otitis media (18). In a previous study, PAO1, a PAO1*ΔwspF* strain with high expression of c-di-GMP and a PAO1/*p_lac_-yhjH* strain with low expression of c-di-GMP were used to successfully construct three PA-BF models of SA (19). Owing to the excessive use of antibiotics, PA has developed high resistance to antibiotics, which poses difficulties for clinical anti-infective treatment. Combination therapy has become a research hotspot in anti-infective treatment (20, 21) and can alleviate the pressure of clinical treatment. Daphnetin (DAP) is a traditional Chinese medicine and a natural coumarin derivative that can inhibit the growth of *Staphylococcus aureus (* 22), inhibit the BF of *Ralstonia solanacearum (* 23), and inhibit and destroy the BF of PA (24). DAP lacks toxicity in animal models (25, 26) and is used clinically as an adjuvant therapy for thromboangiitis obliterans and other occlusive vascular diseases and coronary heart disease. Aminoglycosides are widely used in clinical practice and have broad-spectrum activity against bacteria, including PA. However, with the increasing use of these drugs in clinical practice, the frequency of drug-resistant strains has increased. Because BF formation is an important reason for antibiotic failure/bacterial resistance (4, 5), destruction of biofilm can enhance the effectiveness of antibiotics. It has been reported that DAP has the effect of destroying biofilm (24). This study investigated the effect of DAP combined with tobramycin (TOB) in the treatment of PA-BF infection *in vitro* and *in vivo*, providing a new direction for the clinical treatment of PA-resistant infections.

## 2 Materials and methods

### 2.1 Determination of minimum inhibitory concentration and minimal bactericidal concentration

The MIC of DAP or TOB (Beijing Solaibao Technology Co., Ltd., Beijing, China) or ethylenediaminetetraacetic acid disodium (EDTA-2Na) (Shanghai Test One Chemical Reagent Co., Ltd., Shanghai, China) against the experimental strain was determined using the method described by Belanger et al (27). The PA PAO1 wild-type strain was obtained from the Singapore Centre for Environmental Life Sciences Engineering, Nanyang Technological University, Singapore (28, 29). To prepare the P8 bacterial solution, 1–3 pure colonies were selected and cultured on agar plates overnight. The colonies were then further cultured with Müller-Hinton broth (Qingdao Hopebio Biotechnology Co. Ltd., Qingdao, China) at 37°C and 220 rpm for 14–16 h. The resulting bacterial solutions were centrifuged at 3,000 rpm for 15 min using a centrifuge from HERMLE Labortechnik GmbH (Germany), and any residual media components were removed. The strains were then thoroughly washed three times with phosphate buffer solution (PBS). The concentration of the bacterial solution was adjusted to 1.0 × 10^8^ CFU/mL (OD_600_ = 0.1) using a spectrophotometer (UV-Visible Spectrophotometer T6, Beijing Purse General Instrument Co. Ltd., Beijing, China). DAP (Shanghai Macklin Biochemical Co. Ltd., Shanghai, China) was dissolved in dimethyl sulfoxide (DMSO) (Beijing Solarbio Science Technology Co. Ltd., Guangzhou, China), while TOB was dissolved in normal saline. Different final concentrations of DAP and TOB solutions were prepared (the DMSO solvent was diluted at least 100-fold). Approximately 95 μL of the mixture was added to each well of a 96-well cell culture dish, followed by the addition of 5 μL (OD_600_ = 0.01) of the bacterial solution to each well, with three wells being repeated. In addition, a negative control group (without bacterial solution) and a positive control group (without drugs) were established. The 96-well cell culture dish was placed in a biochemical incubator (HPX-400, Shanghai Yuejin Medical Equipment Co. Ltd., Shanghai, China) at 37°C for 18 h. Finally, the MIC was determined based on the turbidity of the culture. Then, the wells where the concentration was greater than or equal to MIC were incubated at 37°C for an extra 24 h. The concentration at which there was still no visible growth was defined as the MBC (30).The experiment was repeated three times.

### 2.2 Quantitative BF by crystal violet staining *in vitro*

The following method, developed by Haney et al. (31), was used as a reference. A P8 bacterial solution was prepared and 200 μL of the bacterial solution was added to each well of a 96-well cell culture dish. The medium was changed every other day and cultured at 37°C for 72 h. Before treatment, the OD_600_ value of the bacterial solution was measured using a microplate reader. The suspended bacterial solution of each well was then discarded and the BF was gently washed three times with PBS solution, leaving only the adherent growth BF. *In vitro* experiments were divided into the following groups: control group (same amount of medium added without drugs), DMSO group (solvent DMSO diluted 100 times, solvent only), drug group: DAP group (890 μg/mL), TOB group (5.5 μg/mL), EDTA-2Na (2 mg/mL) + TOB (5.5 μg/mL), and DAP (890 μg/mL) + TOB (5.5 μg/mL) group. EDTA-2Na served as a positive control for the experimental treatment. Different groups of 200 μL drugs were added to 96-well cell culture dishes and further cultured at 37°C for 24 h. The supernatant of each well was discarded, and the BF was gently washed three times with PBS solution and then dried at room temperature. A 96-well cell culture dish was stained with 220 μL of 1% crystal violet (100 mL, Beijing Solarbio Science & Technology Co. Ltd., Beijing, China) at room temperature for 30 min. The dye was then poured out, the dish was gently washed three times with pure water and then left to dry (usually for 24 h). A total of 220 μL of 33% glacial acetic acid was added to the 96-well cell culture dish and dissolved at room temperature for 20 min. The OD value was measured at a wavelength of 595 nm using a microplate reader (3020, Thermo Fisher Scientific Oy, Finland).

### 2.3 BF colony count

To determine the concentration of bacteria, the method of He et al. (32) was used. After drug treatment, the supernatant of the original well was removed and the BF was washed three times with PBS solution. Then 100 μL of 0.1% Triton X-100 was added to the well and sonicated for 5 min using a JP-031S sonicator. The resulting bacterial suspension was diluted tenfold using serial dilution and spread evenly on an LB agar plate. After 24 h of incubation at 37°C, the CFU count was performed and the bacterial concentration was calculated. Finally, the results were recorded as logarithmic values with 10 as the base.

### 2.4 Destructive effect of drugs on PA-BF observed using scanning electron microscopy

To prepare the BF carrier, a circle of 1 cm diameter was cut from a 0.3-mm thick polyvinyl chloride (PVC). This circle was soaked in 75% alcohol for one night and then rinsed three times with sterile 0.9% normal saline (NS) to obtain the BF carrier. Next, 1,000 μL of P8 solution was added to a 24-well cell culture dish. Four holes were made and the BF carrier was placed in each hole, ensuring that it was completely immersed in the bacterial solution. The dish was then cultured at 37°C for 72 h. The medium was changed every other day and surface planktonic bacteria were removed by rinsing with sterile PBS water. *In vitro* experiments were conducted using fine groups: control, drug group (890 μg/mL DAP), TOB group (5.5 μg/mL), EDTA-2Na (2 mg/mL) + TOB (5.5 μg/mL), and DAP + TOB group (890 μg/mL DAP + 5.5 μg/mL TOB). One thousand microliters of the respective drug solutions were added to 24-well cell culture dishes and cultured at 37°C for 24 h. After 24 h, the supernatant of each well was discarded and the BF carrier was gently washed three times with PBS solution. It was then placed in 2.5% glutaraldehyde fixative and fixed at 4°C for 12 h. After the removal of the glutaraldehyde, the BF carrier was rinsed with PBS three times for 5 min each time. The BF carrier was dehydrated through a series of ethanol concentrations (30% → 50% → 60% → 70% → 80% → 90%) for 10 min each time. It was then dehydrated twice in 100% ethanol for 10 min each time. The samples were dried in a vacuum freeze dryer for 24 h and then sprayed with gold using the ion sputtering coating method. Finally, the samples were observed and photographed under the SEM. Finally, we performed quantitative analysis of electron microscopy images using Image-Pro Plus 6.0 software (Image-Pro Plus 6.0, Media Cybernetics, USA).

### 2.5 *In vivo* experimental animals and grouping

Healthy and clean New Zealand rabbits, aged 3–4 months, provided by the Experimental Animal Centre of Guangxi Medical University were used in this study. One week before the experiment, each rabbit was placed in an individual cage and provided with water and antibiotic-free rabbit food (80–100 g) at a temperature of 18–25°C with air circulation and 40–70% relative humidity. The rabbits (n = 16) were randomly divided into four groups using a random number table. These groups were the control group (which received the same amount of sterile saline without drugs), the DAP group (which received 890 μg/mL), the TOB group (which received 5.5 μg/mL), and the TOB + DAP group (which received both drugs at the same concentration as the other groups). The study was approved by the Medical Ethics Committee of Guangxi Medical University, and all animal experiments were conducted following the committee’s guidelines.

### 2.6 Rabbit knee joint inoculation

To prepare the bacterial inoculation solution, the P8 bacterial solution was first prepared. This was then diluted 10-fold to a concentration of 10^6^ and set aside. For anesthesia, the rabbits were placed in a rabbit box and slowly injected intravenously with 10% chloral hydrate (5 g/50 mL) X mL (200 mg/kg). For inoculation, the rabbits were fully anesthetized and placed on an operating table in the supine position. Next, the skin of the right knee was prepared with a standard iodophor disinfectant wipe. The knee was then flexed and the patellar ligament and the lateral space between the proximal tibia and distal femur were palpated. Next, 1 mL of the inoculum was injected using a 5 mL syringe. The rabbits were fed routinely for 6 days after inoculation.

### 2.7 Treatment of pyogenic knee arthritis in rabbits

On day 7 of the experiment, two rabbits were randomly selected from each group. Synovial fluid was collected from the infected knee joint for bacterial culture and drug susceptibility testing. The rabbits in each group were injected with one of the following: saline, DAP 890 μg/mL, TOB 5.5 μg/mL, or a combination of DAP 890 μg/mL and TOB 5.5 μg/mL in a 1 mL solution. In addition, rabbits in each group received an intramuscular injection of TOB (10 mg/kg/d) for seven consecutive days to treat any systemic infections.

### 2.8 Anatomy of the rabbit knee joint

On day 14, the rabbits were euthanized using intravenous injection of a lethal dose (200 mg/kg) of 20% chloral hydrate (10 g/50 mL). Samples were then collected by moving the rabbit to the operating table and placing it in the supine position. The right knee joint was disinfected with iodophor and an anterior median incision of approximately 6 cm was made. The skin, subcutaneous tissue, superficial fascia, and deep fascia were incised in sequence. The distal insertion of the patellar ligament was then cut to expose the entire joint space. The results were then photographed. The exudate, debris, and prepatellar synovium in the joint cavity were collected. Additionally, we monitored rabbit body weights and measured serum alanine aminotransferase (ALT) and creatinine levels on days 1, 7, and 14 to evaluate systemic drug toxicity.

### 2.9 Observation and quantification of BF flocs using peptide nucleic acid fluorescence *in situ* hybridization

The PNA-FISH kit (AdvanDx, MA, USA) and the method described in previous literature (33) were used, with some improvements. Sample preparation: We weighed 0.15 g of flakes and rinsed them three times with a sterile PBS solution. We then added 500 μL of PBS solution and homogenized the flocs three times using a grinding machine (sample freezing grinding instrument; Guangzhou Luca Sequencing Instrument Co. Ltd., Guangzhou, China) for 1 min each time at a speed of 70 Hz/s to obtain the homogenate. Slide preparation: Slides were immersed in 75% alcohol for 30 min and then rinsed three times with a sterile PBS solution. We placed 20 μL of homogenized droplets on the slide, evenly coated it with a gun head, and dried it. Then, 10 μL droplets of the *Pseudomonas aeruginosa*-specific PNA probe were placed on the slide in the dark, covered with coverslips, and sealed with rubber. The slides were placed in a humidity box and hybridized in an incubator at 55°C for 60 min. We then removed the rubber and coverslip in the dark, immersed the slide in pure water at 55°C for 30 min, and dried it. We added 10 µL of DAPI staining solution for 15 min. Finally, we used a fluorescence microscope (EVOS FL Auto 2, Invitrogen by Thermo Fisher Scientific, USA) to observe and analyze the image acquisition. After imaging, we used analysis software (Image-Pro Plus 6.0, Media Cybernetics, USA) to measure the red fluorescence area in each film (to select the value of the maximum red area).

### 2.10 Flocs colony count

The 0.15 g flocs were washed three times with a sterile PBS solution. Then 500 µL of the sterile PBS solution was added. The flocs were homogenized three times for 1 min at a speed of 70 Hz/s using a grinding machine. The colonies of each sample were then counted using a continuous tenfold dilution method. Finally, the results were recorded as Xg (CFU/mL) based on the weight, bacterial load, and fluid volume of each sample.

### 2.11 Histopathological examination of the rabbit knee joint using hematoxylin and eosin (H&E) staining

The knee joint tissues were cut to a size of 5 × 5 × 2 mm and fixed in 10% formalin solution for 24 h. It was then rinsed in running water for 24 h. The tissue was then dehydrated using 85% alcohol once, 95% alcohol twice, anhydrous alcohol twice, anhydrous alcohol and xylene (1:1) once, and pure xylene twice (each for 10 min). The next step was wax immersion and embedding. The same amount of paraffin wax was added, and the tissue was immersed in a 50% paraffin-xylene solution for 10 min, followed by a 100% paraffin solution for a further 10 min. The tissue was immersed in wax at 52–56°C for 3 h and then embedded in wax at 60°C. In the sectioning step, the tissue was sectioned (thickness 5 µm), developed, and patched at 42°C for 30 min and baked at 60°C for 12 h. The tissue was then stained and destained with xylene twice (each for 5 min), 95% ethanol twice (each for 3 min), 80% ethanol once (for 3 min), distilled water once (for 1 min), H&E staining for 5 min, color separation with 1% hydrochloric acid-ethanol for 30 s, 0.5% eosin staining for 1 min, distilled water once (for 1 min), anhydrous ethanol for 5 min, and finally dried at room temperature and sealed with neutral gum. The tissues were observed under a fluorescence microscope, and images were captured using an Eclipse Ci-L microscope (Nikon, Japan).

## 3 Statistical analysis

Graph Pad Prism 8.0 software was used to map and analyze the data. The measurement data were expressed as Mean ± SD. One-way analysis of variance (ANOVA) was used to compare the means of normal distribution among multiple groups. LSD test was used for comparison between groups. Rank sum test was used for non-normal distribution. P value < 0.05 was considered statistically significant.

## 4 Results

### 4.1 Drug MIC and minimum bactericidal concentration

The MIC values against PA were as follows: 890 µg/mL for DAP, 2 mg/mL for EDTA-2Na, and 2.75 µg/mL for TOB. The corresponding MBC for TOB was 5.5 µg/mL.

### 4.2 Quantitative results of BF in different treatment groups *in vitro*

**Figure 1:**
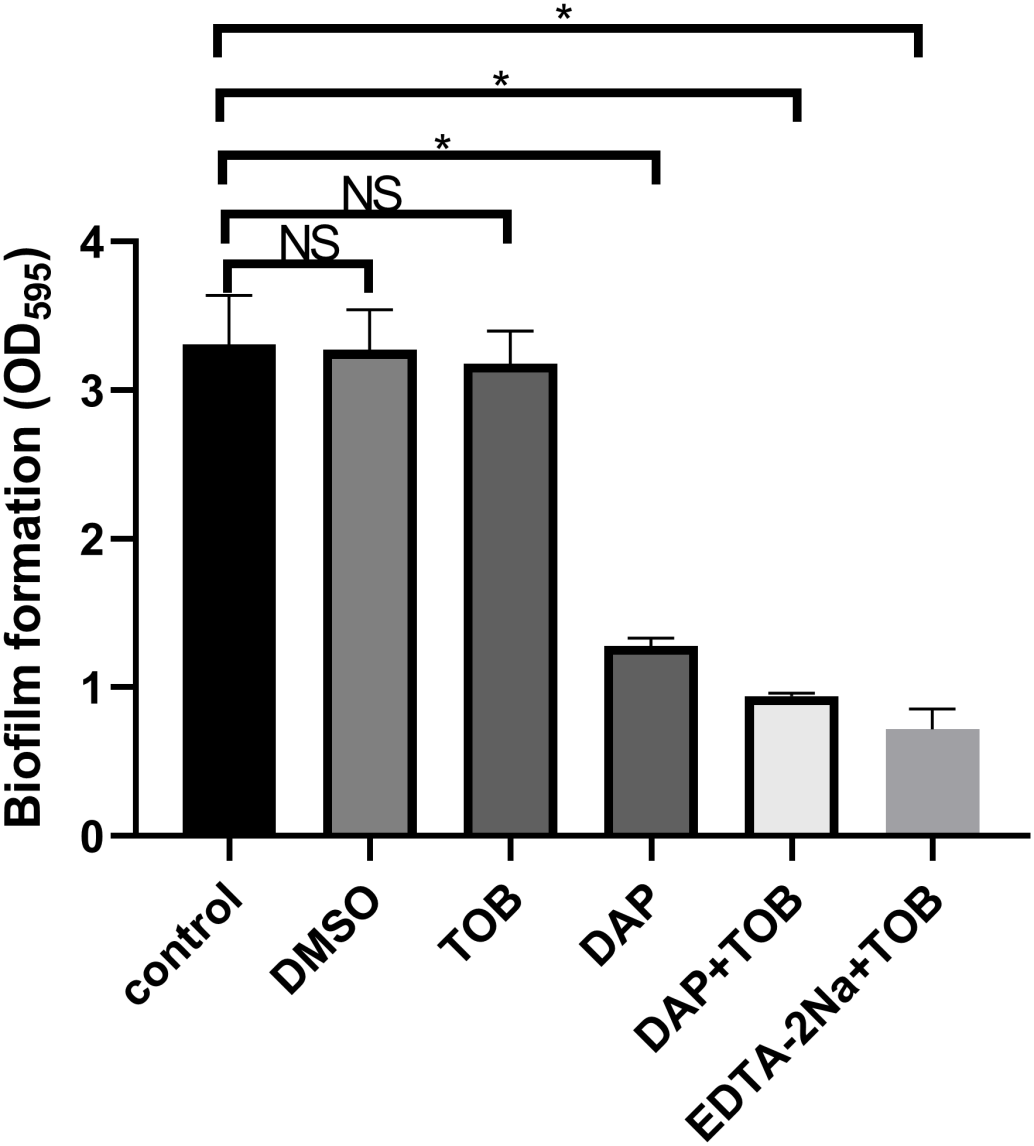
Quantitative results of PA-BF after 24 h of intervention in each group were detected using crystal violet staining *in vitro*. DMSO, dimethyl sulfoxide; TOB, Tobramycin; DAP, Daphnetin; EDTA-2Na, ethylenediaminetetraacetic acid disodium; NS showed no statistical significance compared with the control group, P > 0.05. * shows that compared with the control group, with statistical significance of P < 0.05.

There was no difference in bacterial growth among the groups before treatment, as shown in **Supplementary Material** named “before treatment.” PA-BF was significantly reduced in the DAP monotherapy, EDTA-2Na (2 mg/mL) + TOB (5.5 μg/mL), and DAP (890 μg/mL) + TOB (5.5 μg/mL) treatment groups compared to the control and TOB monotherapy groups (P < 0.05). However, no statistical difference was observed between the EDTA-2Na+TOB and DAP+TOB combination groups. Consistent with **Figure 1**, the DMSO solvent control, TOB alone, and untreated control groups showed comparable biofilm levels.

### 4.3 Number of viable bacteria in BFs of different treatment groups *in vitro*

**Figure 2:**
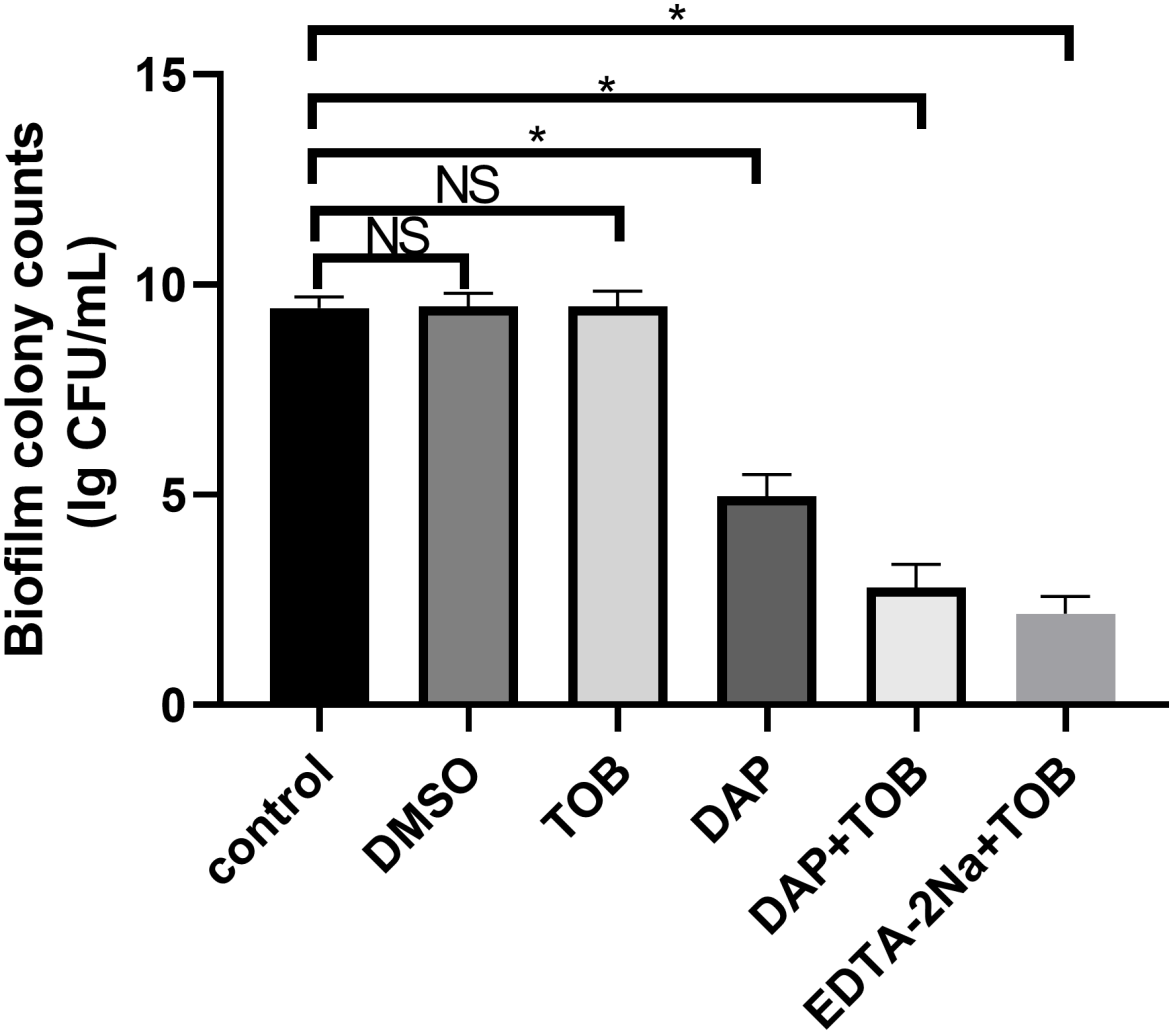
Quantitative results of PA-BF after 24 h of intervention in each group were detected using colony counting. DMSO, dimethyl sulfoxide; TOB, Tobramycin; DAP, Daphnetin; EDTA-2Na, ethylenediaminetetraacetic acid disodium; NS showed no statistical significance compared with the control group, P > 0.05. * shows that compared with the control group, with statistical significance of P < 0.05.

No significant reduction in viable bacterial counts within BF was observed following 24 h treatment with 5.5 μg/mL TOB alone. In contrast, treatments with DAP monotherapy, EDTA-2Na+TOB, and TOB+DAP all demonstrated significantly lower viable bacterial counts compared to the control group (P < 0.05). The DMSO solvent control, TOB monotherapy, and untreated control groups showed comparable bacterial viability (**Figure 2**).

### 4.4 Electron microscopic observation of BF morphology in different treatment groups *in vitro*

**Figure 3:**
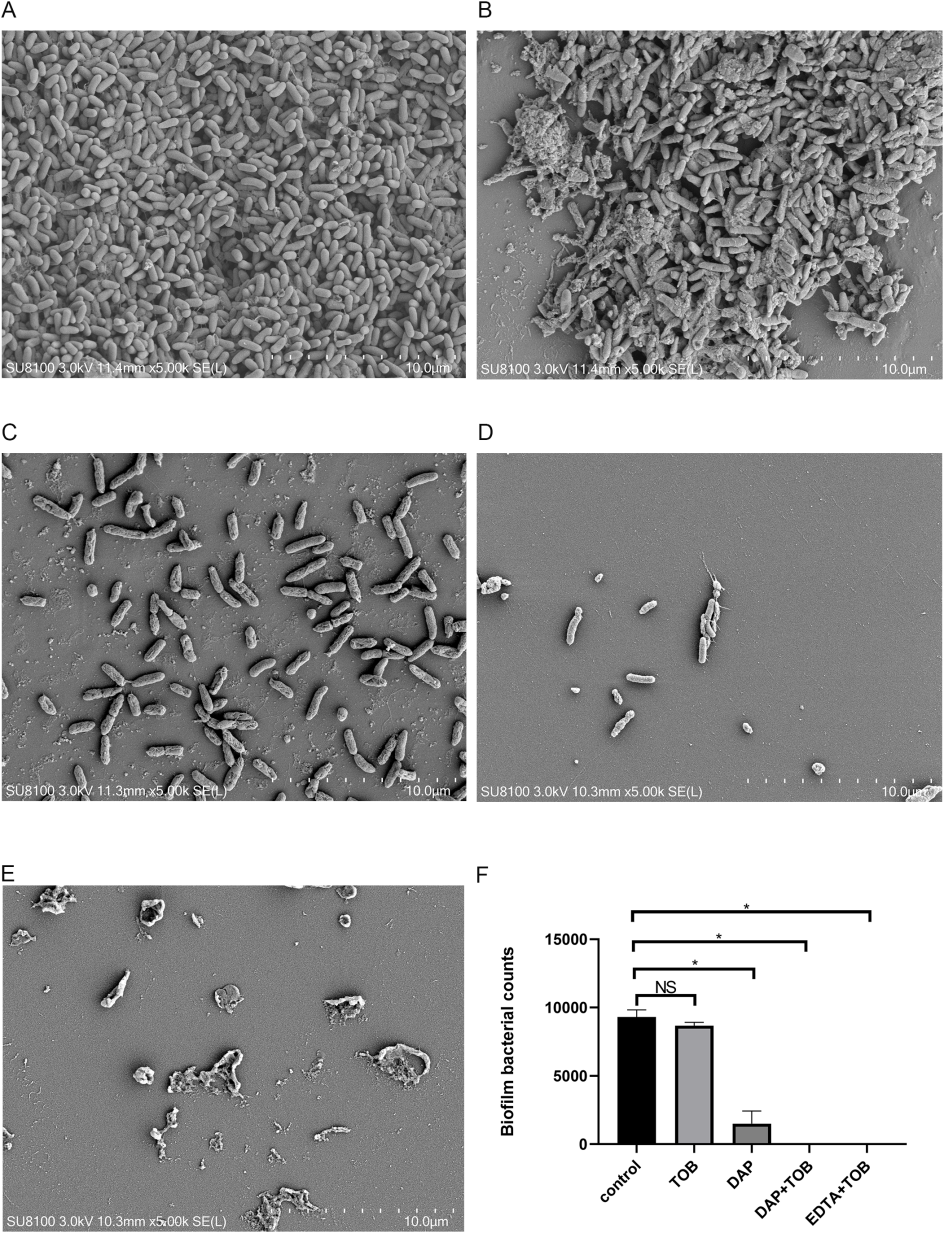
SEM observation of PA-BF after 24 h of intervention in each group. **(A)** control group; **(B)** TOB group; **(C)** DAP group; **(D)** TOB + DAP group. **(E)** EDTA-2Na+TOB; **(F)** Quantitative electron microscopy analysis. TOB, Tobramycin; DAP, Daphnetin; EDTA-2Na, ethylenediaminetetraacetic acid disodium. NS showed no statistical significance compared with the control group, P > 0.05. * shows that compared with the control group, with statistical significance of P < 0.05.

In **Figure 3A**, the control group showed a significant amount of dense BF formation on the carrier surface. They also secreted some mucilaginous substances that were tightly associated with each other, and the pathogen PA was tightly wrapped in the BF. In **Figure 3B**, the single TOB group showed a similar amount of BF on the carrier surface, however, the number of PA decreased slightly, and it could be considered that the planktonic cells were killed by TOB. In the DAP alone group (**Figure 3C**), the BF on the surface of the carrier reduced significantly, but there were still several bacteria attached to the carrier surface and some BF-like substances on the bacterial surface. In contrast, carriers in both the DAP+TOB group (**Figure 3D**) and EDTA-2Na+TOB group (**Figure 3E**) showed complete absence of biofilm structures, with only sparse individual bacteria observed. Quantitative electron microscopy analysis (**Figure 3F**) confirmed significant reductions in biofilm bacterial counts for the DAP monotherapy, DAP+TOB, and EDTA-2Na+TOB treatment groups compared to controls.

### 4.5 General condition of the rabbits *in vivo* and anatomical observation of the knee joint

**Figure 4:**
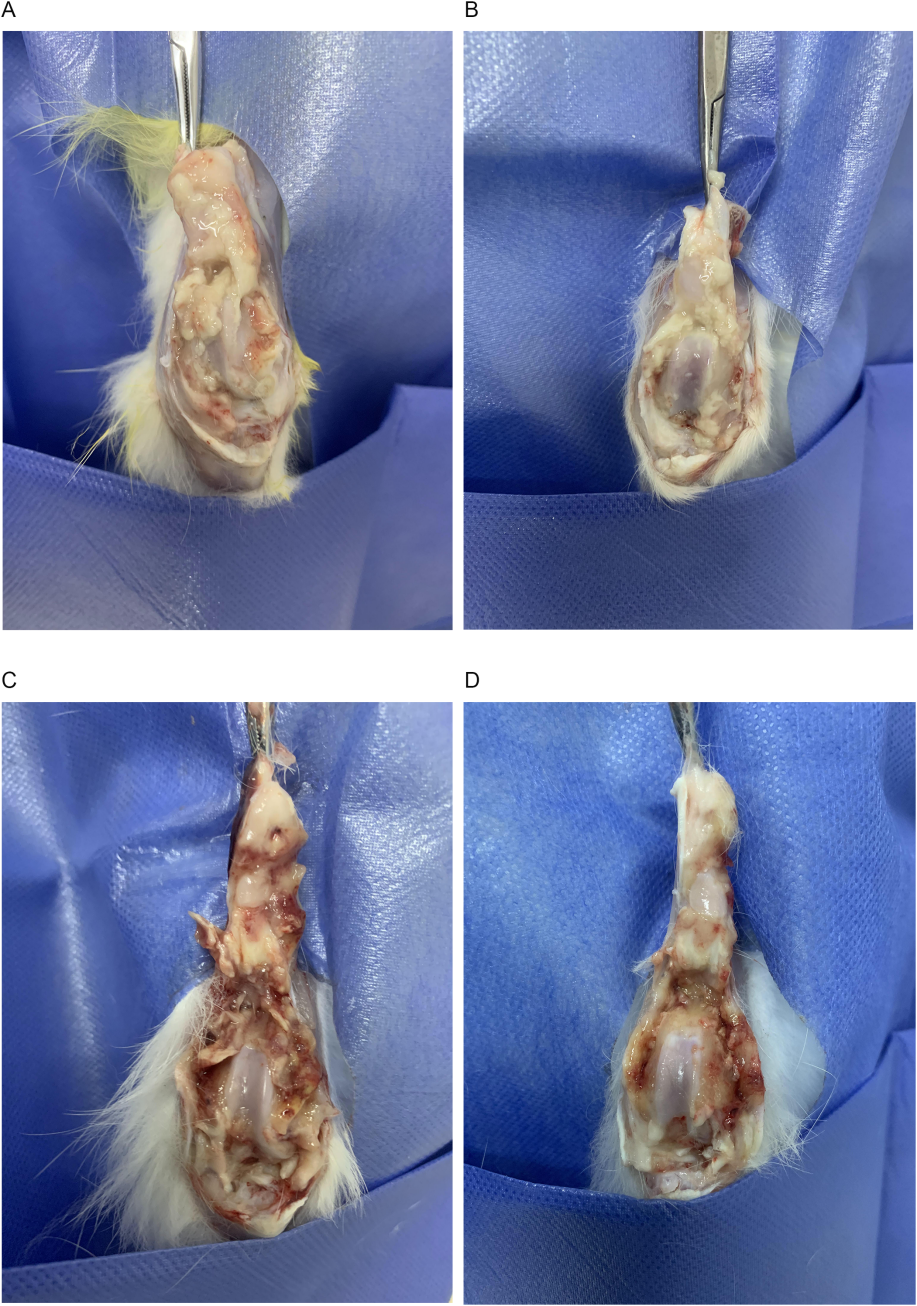
Observation of gross anatomy of rabbit knee joint on day 4 after *in vivo* treatment. **(A)** control group; **(B)** TOB group; **(C)** DAP group; **(D)** TOB + DAP group. TOB, Tobramycin; DAP, Daphnetin.

On day 14 of the experiment, the fluid from the affected knee joint was collected for bacterial culture and drug susceptibility testing. The results showed that PA was infected and sensitive to TOB, confirming the success of the suppurative knee joint infection model. All 16 rabbits survived throughout the experiment. In the control and DAP groups, the rabbits gradually lost their appetite and activity during the treatment. Their knee joints became swollen and painful, and had flexion contractures. In contrast, the rabbits in the TOB and TOB + DAP groups had better appetite and activity than those in the control group, with the TOB + DAP group being the best. When the knee joints were examined, the control and DAP groups had a large amount of purulent secretions and debris in the joint cavity and severe congestion of the synovium. Meanwhile, the TOB group had a moderate amount of purulent secretions and debris, with moderate synovial congestion. Finally, the TOB + DAP group had a small amount of purulent secretions and flakes with a small amount of synovial congestion, as shown in **Figure 4**.

### 4.6 The results of PA-BF flocculation were observed and quantified using PNA-FISH

**Figure 5:**
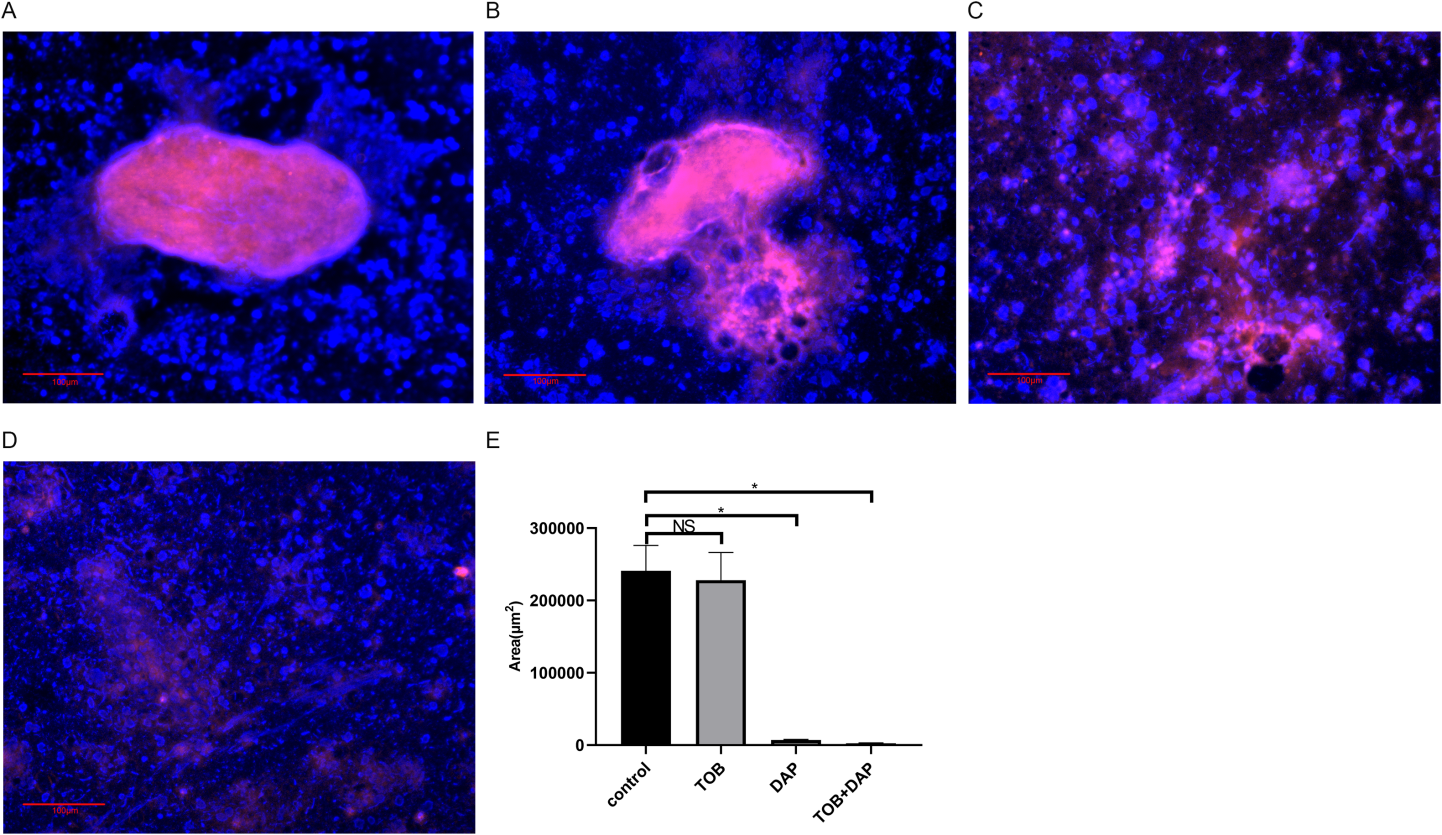
Observation of BF in floccules using PNA-FISH on day 4 after *in vivo* treatment. The PNA-FISH kit uses PA-specific probes (red) and non-specific nucleic acid staining DAPI (blue) to identify BF (× 40). **(A)** control group; **(B)** TOB group; **(C)** DAP group; **(D)** DAP and TOB group. TOB, Tobramycin; DAP, Daphnetin; NS showed no statistical significance compared with the control group, P > 0.05. * shows that compared with the control group, with statistical significance of P < 0.05.

In **Figures 5A, B**, representing the control and the TOB groups, respectively, a significant amount of red fluorescence (PA) was surrounded by blue fluorescence (matrix), indicating the presence of BF aggregates embedded in the matrix. However, in **Figures 5C, D**, representing the DAP and DAP + TOB groups, respectively, the amount of BF was less than that of the control and TOB groups. The combined group had the least amount of BF. After analyzing the images using Image-Pro Plus 6.0 software, it was found that the amount of BF in the control and the TOB groups was significantly more than that in the DAP and the combined groups (P < 0.05), as shown in **Figure 5E**.

### 4.7 Results of *in vivo* flocculation colony counting

**Figure 6:**
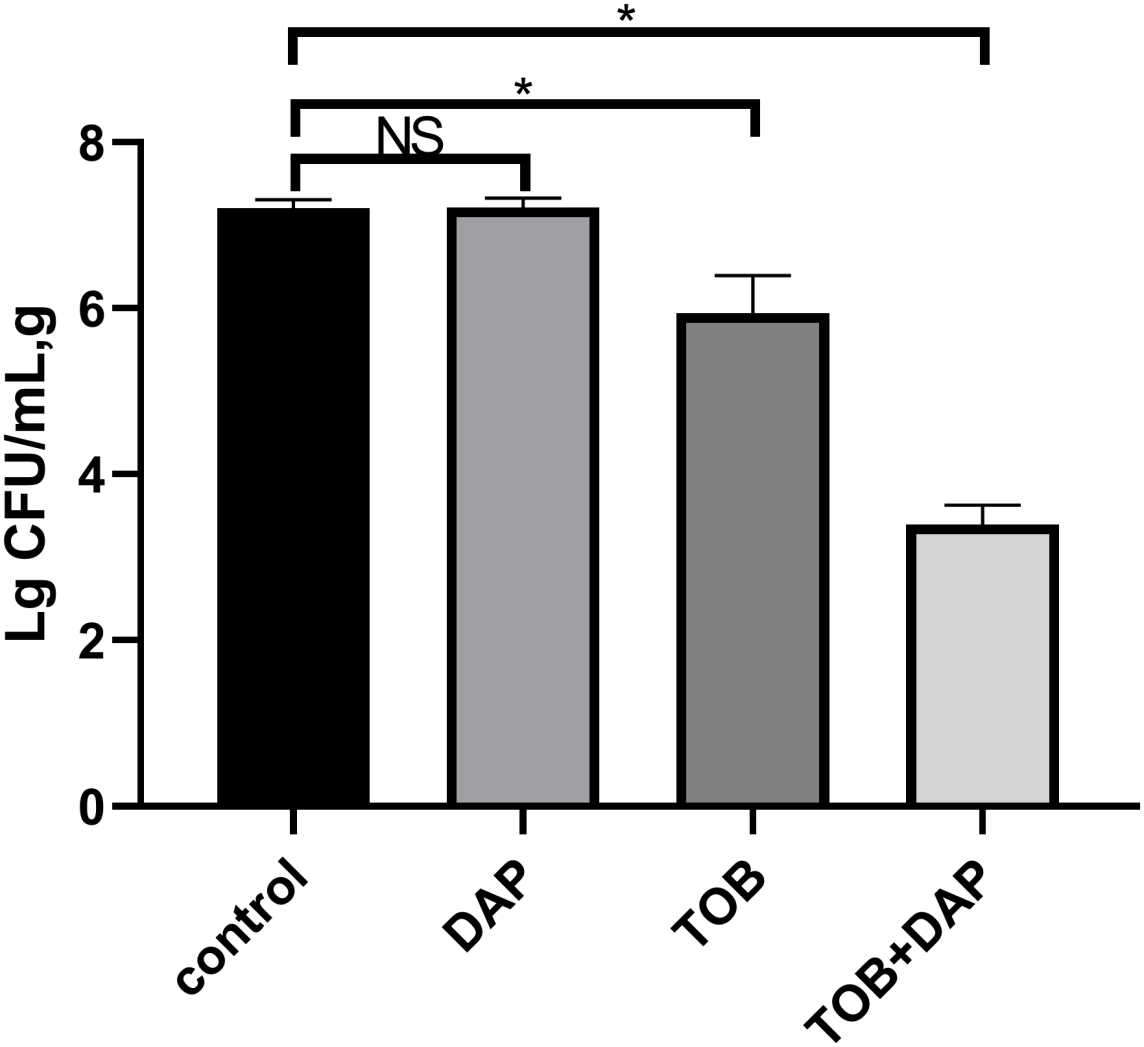
The results of flocculent colony count on day 4 after *in vivo* treatment. TOB, Tobramycin; DAP, Daphnetin, NS showed no statistical significance compared with the control group, P > 0.05. * shows that compared with the control group, with statistical significance of P < 0.05.

The results of the experiment were as follows: the colony counts of each group were expressed as Lg CFU/mL, g, and are presented as mean ± SD. The control group had a count of 7.208 ± 0.11, the DAP group had a count of 7.215 ± 0.12, the TOB group had a count of 5.945 ± 0.45, and the TOB + DAP group had a count of 3.465 ± 0.18. There was no significant difference between the DAP and the control groups (P > 0.05). However, there was a significant difference between the other groups and the control group (P < 0.05), as shown in **Figure 6**.

### 4.8 Results of H&E staining of rabbit knee joint histopathology *in vivo*

**Figure 7:**
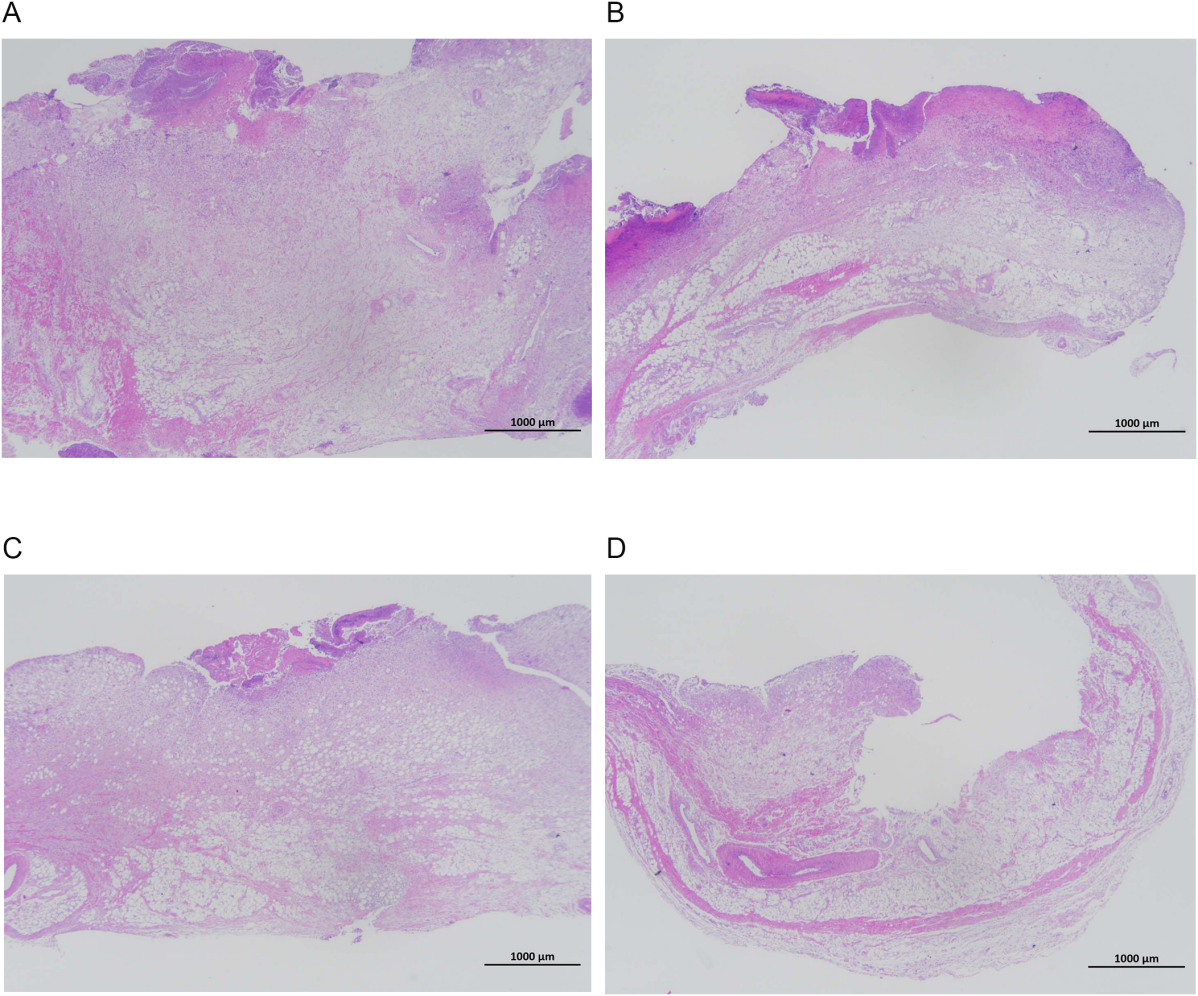
Pathological changes in the synovium of the rabbit knee joint after 4 days of treatment (H&E staining, × 20). **(A)** control group; **(B)** DAP group; **(C)** TOB group; **(D)** TOB + DAP group. TOB, Tobramycin; DAP, Daphnetin.

**Figure 8:**
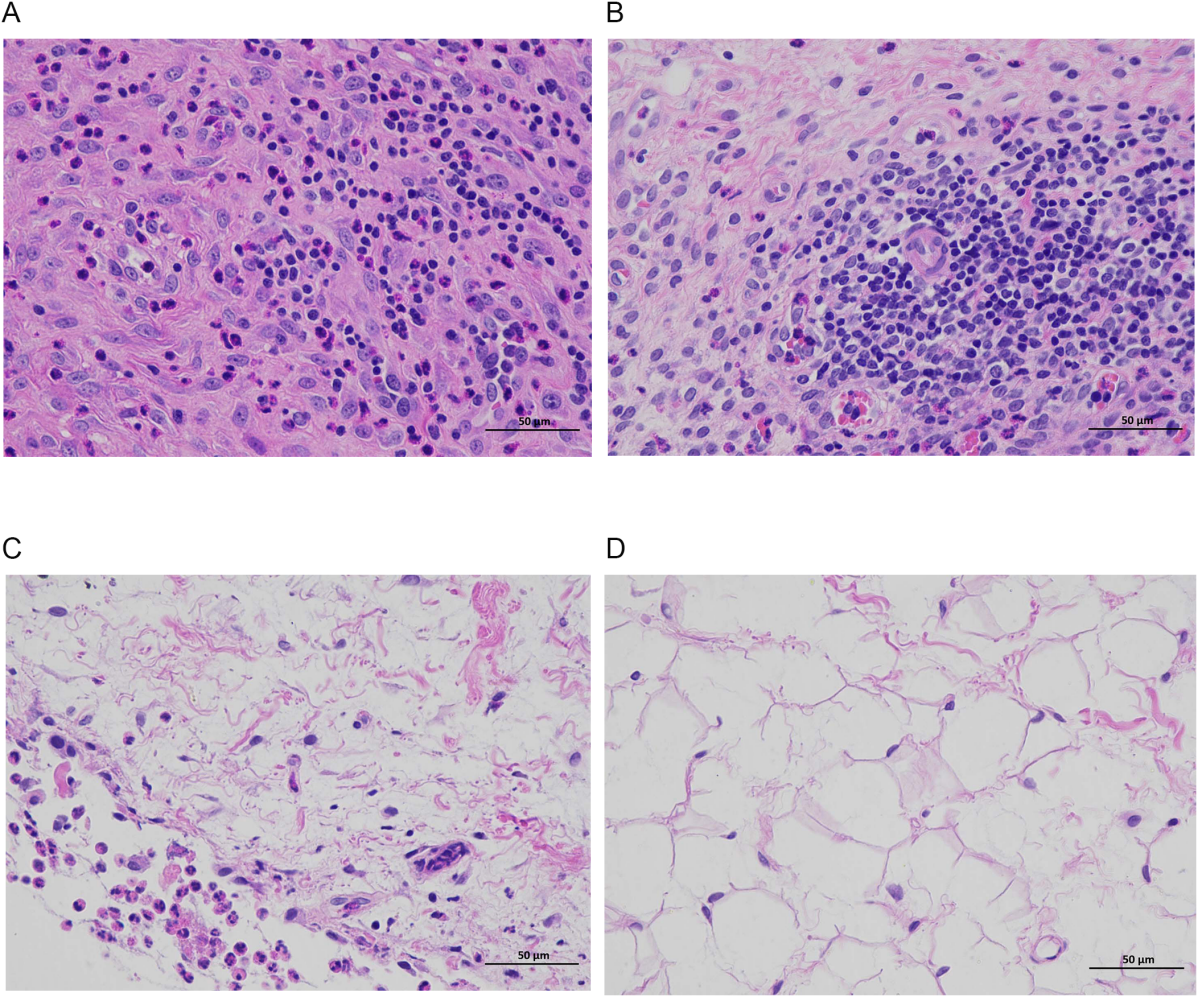
Pathological changes in the synovium of the rabbit knee joint after 4 days of treatment (H&E staining, × 400). **(A)** control group; **(B)** DAP group; **(C)** TOB group; **(D)** TOB + DAP group. TOB, Tobramycin; DAP, Daphnetin.

**Figure 9:**
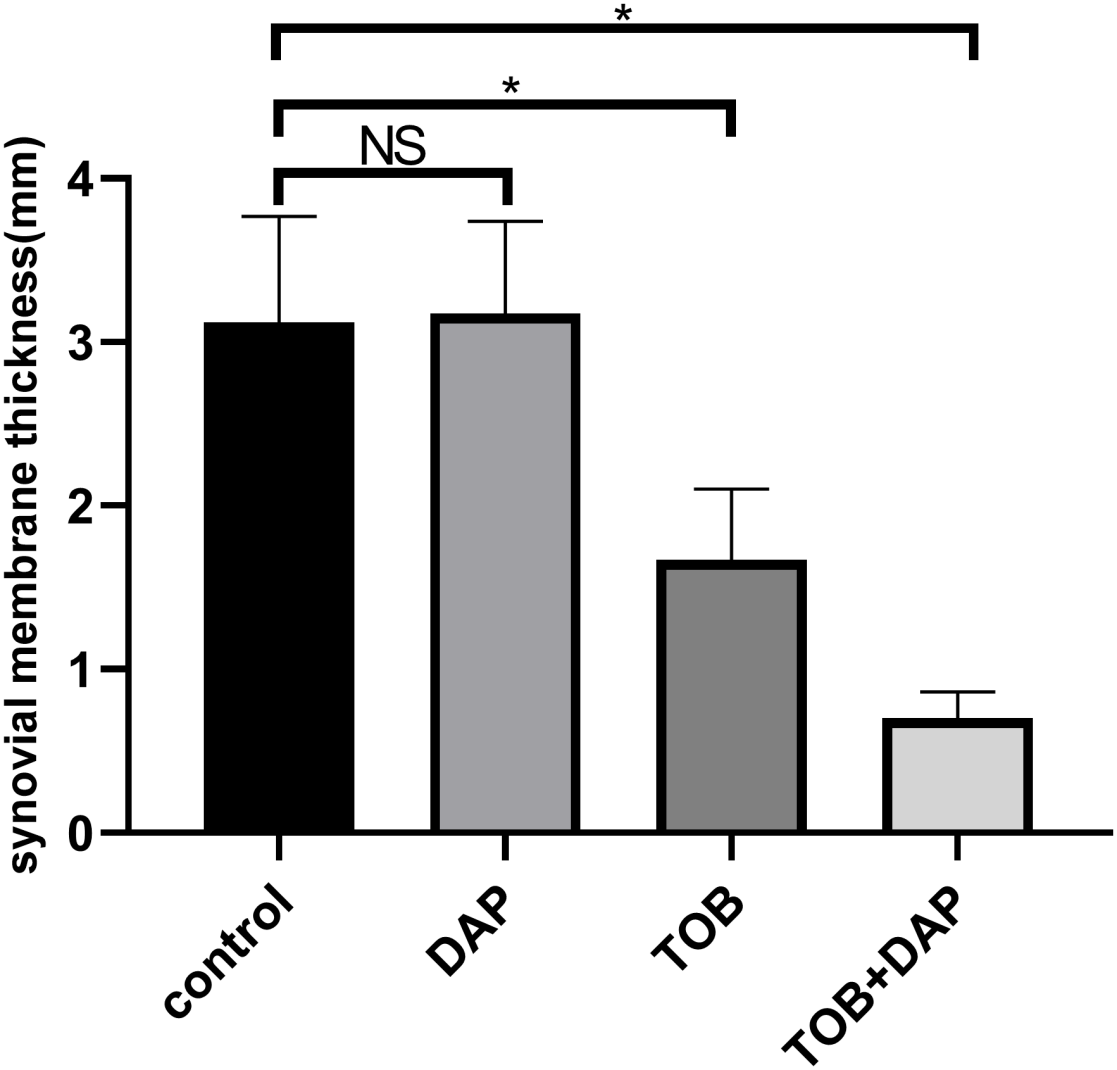
The thickness of the anterior patellar synovium of the rabbit knee was measured under the H&E staining microscope on day 4 after *in vivo* treatment. TOB, Tobramycin; DAP, Daphnetin; NS showed no statistical significance compared with the control group, P > 0.05. * shows that compared with the control group, with statistical significance of P < 0.05.

**Figures 7**, **8** show that the control and DAP groups had high levels of inflammatory cell infiltration. Moderate inflammatory cell infiltration was observed in the TOB group, and mild inflammatory cell infiltration was observed in the TOB + DAP group. The thickness of the prepatellar synovium was measured in each group (mean ± SD), as shown in **Figure 9**. The control group had a thickness of 3.120 ± 0.65 mm, the DAP group had a thickness of 3.176 ± 0.56 mm, the TOB group had a thickness of 1.668 ± 0.43 mm, and the TOB + 0.890 µg/mL DAP group had a thickness of 0.922 ± 0.11 mm. There was no significant difference between the DAP and control groups (P > 0.05). However, there was a significant difference between the other groups and the control group (P < 0.05).

### 4.9 Systemic toxicity assessment in rabbits

**Figure 10:**
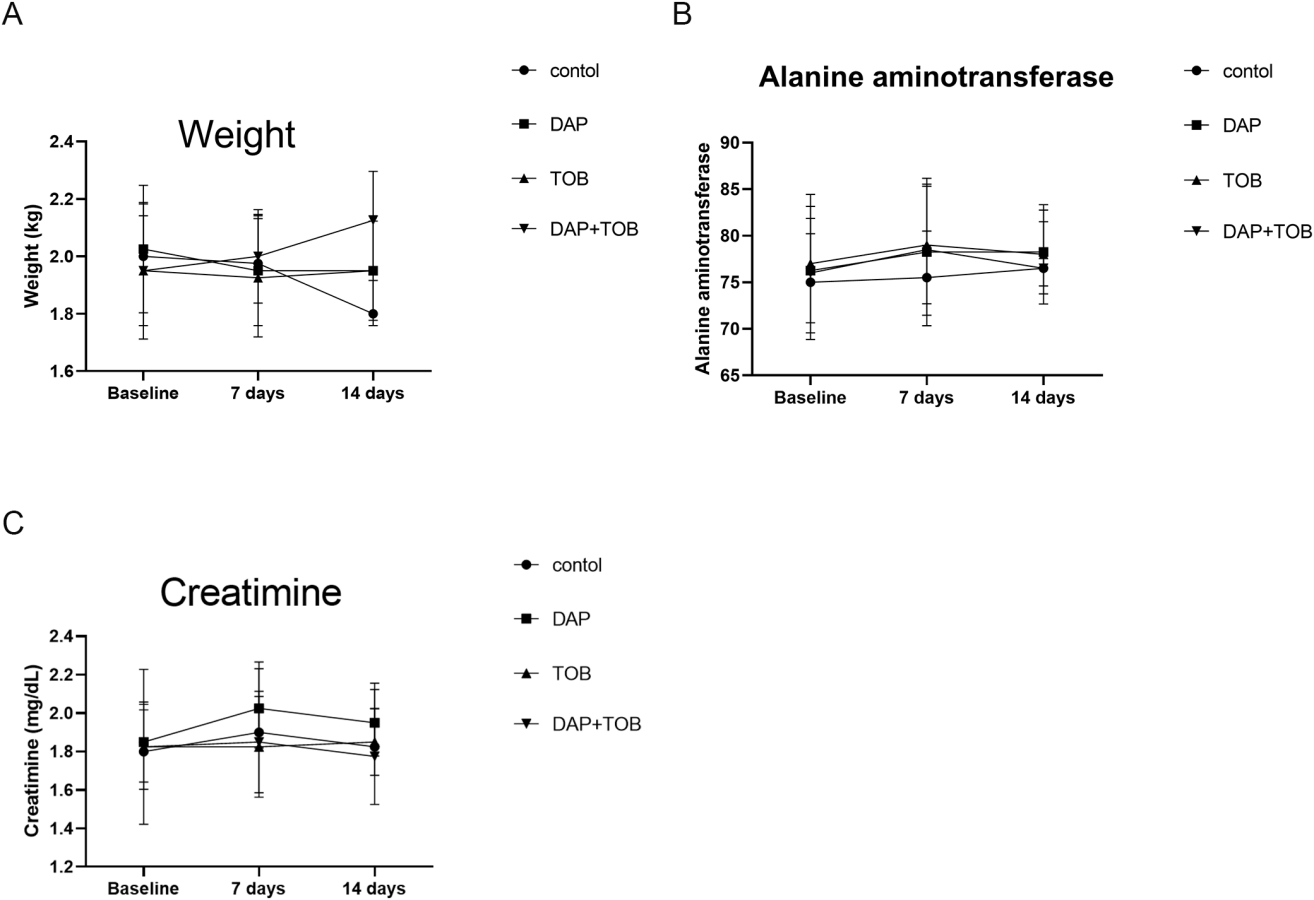
Rabbit body weight and serum biomarkers (ALT, Creatinine) at Days 1, 7, and 14. TOB, Tobramycin; DAP, Daphnetin.

All rabbits survived the experimental period. Body weights (**Figure 10A**) and serum alanine aminotransferase (ALT) (**Figure 10B**) and creatinine levels (**Figure 10C**) were measured on days 1, 7, and 14. Results indicated: Weight changes: Control group showed minor weight reduction, while treatment groups remained unaffected. Biomarkers: All creatinine and ALT values fluctuated within physiologically normal ranges without significant alterations. No treatment-related systemic toxicity was observed.

## 5 Discussion

Septic arthritis (SA) is a type of arthritis caused by direct infection with purulent bacteria, resulting in joint destruction and loss of function (1, 2). One of the major challenges in the treatment of SA is bacterial resistance caused by antibiotic tolerance mechanisms such as the formation of pathogenic bacterial BF (4, 5). PA is a common bacterium that causes SA in hospitals and is a highly invasive pathogen that poses a threat to public health due to its virulence factors, BFs, and multidrug resistance (34). The emergence of multidrug-resistant bacteria has made clinical anti-infective treatment more challenging, leading to the investigation of combination therapy as a potential solution (20, 21). In this context, 7,8-dihydroxycoumarin (DAP) is a traditional Chinese medicine that has anti-inflammatory (22), anti-osteoarthritic (25), and inhibitory effects on *R. solanacearum* BF (23) and PA-BF destruction (24). In a previous study, the researchers successfully constructed three PA-BF models of SA using PAO1, PAO1*ΔwspF* strain with high expression of c-di-GMP, and PAO1/*p_lac_-yhjH* strain with low expression of c-di-GMP (19). In this study, the researchers investigated the inhibitory effect of DAP combined with TOB on PA-BFs both *in vitro* and *in vivo*. Inhalation of TOB is the preferred maintenance therapy for chronic PA infection (35). However, the emergence of resistance to this antibiotic—including among PA strains—has prompted efforts to enhance its efficacy. Researchers now explore liposomal formulations that chemically conjugate or physically encapsulate TOB with other bioactive molecules (36). Given high resistance rates, TOB is unsuitable as empirical first-line therapy for severe Gram-negative bacterial infections, particularly in high-resistance regions or high-risk patients. Compared to macrolides, colistin, polymyxin B, β-lactams, and fluoroquinolones, TOB demonstrates superior bactericidal activity with lower nephrotoxicity and neurotoxicity. These properties justify its selection as the antibiotic for this study.

The study found that the combination of DAP and TOB had a positive antibacterial effect on PA. Previous research has also shown that quinazolinone (QZN) 34 combined with TOB has antibacterial activity against PA (37), which supports the conclusions of this study. Although commonly used antibiotics, such as TOB, are effective against PA in the free state, they are not effective against PA in the BF state. However, the combination of TOB and DAP showed a synergistic inhibitory effect against BF bacteria. This could be due to the disruption of the BF structure by DAP, the enhanced penetration ability of TOB into BF, and the improved killing ability of TOB against BF bacteria. In this study, it was observed that the number of viable bacteria in BF was significantly lower after DAP + TOB acted on the BF of PA *in vitro* for 24 h or *in vivo* for 7 days. DAP has the effect of destroying the BF, which is consistent with previous studies. In addition, electron microscopy results showed that the proportion of dead bacteria was higher for DAP + TOB treatment than for the control and TOB groups. The killing effect of DAP alone on PA was weak, but it significantly destroyed the structure of BF. TOB was still effective against PA, and when combined with DAP, its killing effect on PA was significantly improved. DMSO (DAP solvent) diluted 100 times did not affect the growth of PA.

The results of the *in vivo* experiments showed that the control and DAP groups had a numerous of purulent secretions, floccules, severe synovial hyperemia, more bacterial colony counts, and extensive inflammatory cell infiltration on pathological examination. In contrast, the TOB + DAP group had the mildest signs of infection, the lowest bacterial colony count of flocules, and the least inflammatory cell infiltration on pathological examination. This indicates that a concentration of 0.890 mg/mL of DAP alone had no bactericidal effect, but had a synergistic effect with TOB. DAP destroys the PA-BF, which increases the sensitivity of TOB to PA, consistent with the *in vitro* study results. Current antibacterial drugs are not effective against PA-BF infection and it is usually necessary to combine them with other QS inhibitors to enhance their effect. For example, the use of curcumin in combination with sub-inhibitory concentrations of azithromycin, ceftazidime, or ciprofloxacin has been observed to enhance the inhibitory effect on PA-BF formation (38, 39). Li et al. (40) demonstrated the synergistic effect of berberine as a QS inhibitor and azithromycin on PA. Christensen et al. (41) showed that garlicene, furanone C-30, and horseradish juice extracts as QS inhibitors combined with TOB had a synergistic anti-PA-BF effect. Recent studies have also shown that phytochemicals, such as cinnamic acid (42), 6-methylcoumarin (43), sesamin, and sesamolin (44) attenuate QS-regulated virulence and BF formation. They protect *C. elegans* from PA infection and prolong its lifespan. These results are similar to our *in vivo* findings. And our vivo toxicity assessment in rabbits demonstrated no significant effects on body weight or hepatic and renal function biomarkers. However, the sample size of this part of the study is limited, and further studies are needed to verify the effect of DAP combined with TOB on PA-BF infection and to explore the underlying mechanism.

In conclusion, DAP + TOB treatment showed a promising therapeutic effect on PA-BF infection. It is expected that this treatment will be considered a viable option for the treatment of PA-BF infection, paving the way for the potential clinical application of DAP and opening new avenues for clinical anti-infective therapy.

## Data Availability

The original contributions presented in the study are included in the article/**Supplementary Material**. Further inquiries can be directed to the corresponding authors.

## Supplementary material

Supplementary material 1 Quantitative results of PA growth for 72 h detected using a microplate reader. Note: NS showed no statistical significance compared with the control group, P > 0.05. * shows that compared with the control group, with statistical significance of P < 0.05.
