# Supplementary figures and images for: Effect of daphnetin combined with tobramycin on Pseudomonas aeruginosa biofilm infection in vitro and in vivo
Source: Front Immunol. 2025 Aug 8;16:1648096. doi: 10.3389/fimmu.2025.1648096 (PMC12370681; doi:10.3389/fimmu.2025.1648096)

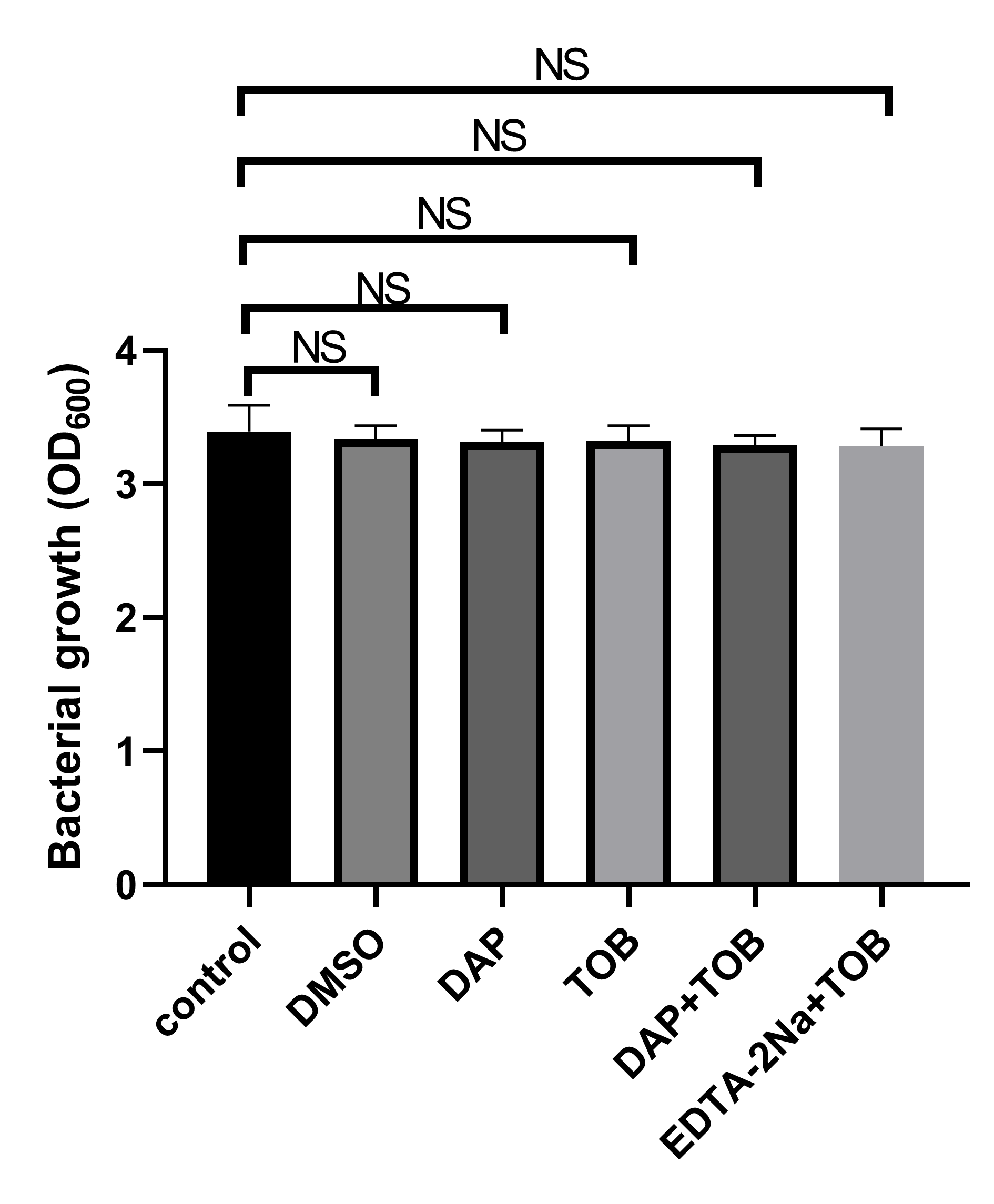

Supplement: Supplementary material 1 — Quantitative results of PA growth for 72 h detected using a microplate reader. Note: NS showed no statistical significance compared with the control group, P > 0.05. * shows that compared with the control group, with statistical significance of P < 0.05. [file Image1.tif]
